# Supplementary material for: Psychological interventions for generalized anxiety disorder: Effects and predictors in a naturalistic outpatient setting
Source: PLoS One. 2023 Mar 10;18(3):e0282902. doi: 10.1371/journal.pone.0282902 (PMC10004605; doi:10.1371/journal.pone.0282902)
Supplement: S1 Table — (DOCX) [file pone.0282902.s001.docx]

**S1 Table. Pooled correlations between all measures.**

| **Variable** | 1 | 2 | 3 | 4 | 5 | 6 | 7 | 8 | 9 | 10 | 11 | 12 | 13 | 14 | 15 | 16 | 17 |
| --- | --- | --- | --- | --- | --- | --- | --- | --- | --- | --- | --- | --- | --- | --- | --- | --- | --- |
| 1. PSWQ pre | — |  |  |  |  |  |  |  |  |  |  |  |  |  |  |  |  |
| 2. PSWQ post | .51^**^ | — |  |  |  |  |  |  |  |  |  |  |  |  |  |  |  |
| 3. MCQ-NEG pre | .47** | .08 | — |  |  |  |  |  |  |  |  |  |  |  |  |  |  |
| 4. MCQ-NEG post | .33* | .63** | .02 | — |  |  |  |  |  |  |  |  |  |  |  |  |  |
| 5. MCQ NEG Δ | .13 | -.37** | .74** | -.66** | — |  |  |  |  |  |  |  |  |  |  |  |  |
| 6. MCQ-POS pre | .29* | .25 | .03 | .12 | -.06 | — |  |  |  |  |  |  |  |  |  |  |  |
| 7. MCQ-POS post | .24 | .40** | -.25 | .35** | -.43** | .36** | — |  |  |  |  |  |  |  |  |  |  |
| 8. MCQ POS Δ | .13 | -.02 | .21 | -.12 | .24 | .77** | -.32* | — |  |  |  |  |  |  |  |  |  |
| 9. IUS pre | .50** | .27* | .41** | .04 | .28* | .45** | .08 | .39** | — |  |  |  |  |  |  |  |  |
| 10. IUS post | .54** | .55** | .07 | .34** | -.18 | .40** | .42** | .12 | .56** | — |  |  |  |  |  |  |  |
| 11. IUS Δ | .05 | -.22 | .40** | -.28* | .49** | .11 | -.32* | .33* | .59** | -.34** | — |  |  |  |  |  |  |
| 12. BDI pre | .35** | .17 | .29* | .07 | .18 | .32* | .16 | .21 | .40** | .37** | .10 | — |  |  |  |  |  |
| 13. BDI post | .24 | .28* | .01 | .48** | -.31* | .37** | .26 | .19 | .16 | .32* | -.12 | .30* | — |  |  |  |  |
| 14. GSI pre | .47** | .26* | .39** | .20 | .15 | .36** | .24 | .20 | .37** | .32* | .12 | .72** | .25 | — |  |  |  |
| 15. GSI post | .31* | .41** | .03 | .45** | -.29* | .32* | .27* | .14 | .09 | .38** | -.27 | .33* | .72** | .28* | — |  |  |
| 16. Age | -.22 | -.11 | -.12 | -.03 | -.07 | -.06 | .02 | -.08 | -.16 | -.03 | -.15 | -.17 | -.08 | -.07 | -.16 | — |  |
| 17. Sex^†^ | .13 | .30* | .15 | .05 | .08 | .07 | -.07 | .12 | .15 | .20 | -.03 | .19 | .12 | .02 | .15 | -.06 | — |
| 18. Comorbidity^‡^ | .11 | .03 | -.11 | -.02 | -.07 | -.14 | .07 | -.20 | -.17 | .03 | -.22 | -.06 | -.20 | -.15 | -.09 | .04 | .04 |

PSWQ = Penn State Worry Questionnaire; MCQ-NEG = negative metacognitive beliefs of the Metacognitions Questionnaire; MCQ-POS = positive metacognitive beliefs of the Metacognitions Questionnaire; IUS = Intolerance of Uncertainty Scale. Δ = change scores.

^†^0 = male, 1 = female; ^‡^0 = no comorbid disorders; 1 = at least one comorbid disorder.

**p* < .05; ***p* < .01.
